# Feasibility of using a hand-held device to characterize tendon tissue biomechanics

**DOI:** 10.1371/journal.pone.0184463

**Published:** 2017-09-06

**Authors:** Sahand Sohirad, David Wilson, Charlotte Waugh, Evan Finnamore, Alexander Scott

**Affiliations:** 1 Centre for Hip Health and Mobility, Vancouver Coastal Health Research Institute, Vancouver, Canada; 2 Department of Orthopaedics, Faculty of Medicine, University of British Columbia, Vancouver, Canada; 3 Department of Physical Therapy, Faculty of Medicine, University of British Columbia, Vancouver, Canada; University of Zaragoza, SPAIN

## Abstract

**Objectives:**

To examine the feasibility of using the MyotonPRO digital palpation device in measuring the transverse stiffness of tendon tissue.

**Design:**

Experimental study.

**Methods:**

The MyotonPRO was used to measure the stiffness and related properties of ballistics gel in comparison with an external materials testing system (PCB electronics). The device was then used to measure the same properties of avian Achilles tendons before and after the removal of the overlying skin and subcutaneous tissue. Next, the test-retest reliability of the Achilles and patellar tendons was determined in humans. Finally, the stiffness of the Achilles tendon was measured before and after competitive running races of varying distances (10, 21 and 42 km, total number of athletes analyzed = 66).

**Results:**

The MyotonPRO demonstrated a high degree of consistency when testing ballistics gel with known viscoelastic properties. The presence of skin overlying the avian Achilles tendon had a statistically significant impact on stiffness (p<0.01) although this impact was of very small absolute magnitude (with skin; 728 Nm ±17 Nm, without skin; Nm 704 Nm ±7 Nm). In healthy adults of normal body mass index (BMI), the reliability of stiffness values was excellent both for the patellar tendon (ICC, 0.96) and the Achilles tendon (ICC,0.96). In the the field study, men had stiffer tendons than women (p<0.05), and the stiffness of the Achilles tendon tended to increase following running (p = 0.052).

**Conclusions:**

The MyotonPRO can reliably determine the transverse mechanical properties of tendon tissue. The measured values are influenced by the presence of overlying skin, however this does not appear to compromise the ability of the device to record physiologically and clinically relevant measurements.

## Introduction

The MyotonPRO (Myoton AS, Tallinn, Estonia) is a handheld, digital palpation device which has been used to measure the mechanical properties of muscles and other soft tissue [1]. Using such a device, characterization of the biomechanical properties of the musculoskeletal system has the potential to help identify and diagnose abnormalities in skeletal tissues on-site and in the field without the need of other highly specialized equipment. For example, areas of increased muscle tone[2] or the response of hypertonic muscle to therapeutic interventions[3] have been reported. The MyotonPRO works by imparting a small mechanical impact to the tissue of interest, perpendicular to the surface of the skin. The tip of the probe is subjected to a constant preload to maintain contact and co-oscillation as the tissue vibrates underneath the skin. An accelerometer linked to the probe generates an acceleration vs. time relation from which various biomechanical characteristics, such as tissue stiffness, can be calculated.[4]

The repeatability and reliability of the device have been tested on various muscles in several intra and inter-session studies,[5–7] but never on tendon tissue to our knowledge. This study aims to conduct an independent assessment by a publically funded, academic research group of the MyotonPRO’s stiffness measurement by (1) testing on phantom material with experimentally verified viscoelastic properties, (2) determining whether or not (and to what extent) the device’s measurements are influenced by the layer of skin over the tissue being measured, (3) examining the test-retest reliability of the MyotonPRO applied to human Achilles and patellar tendons and (4) examining the performance of the device during a field study of tendon stiffness in endurance runners.

## Methods

### Examination of biomechanical parameters

To create simulated soft tissue phantoms, ballistics gels of different consistencies (1:30, 1:20, and 1:10 mass ratio of gelatin to water) were prepared using commercially-produced gelatin powder (Knox Gelatin, Gelita USA Inc. Sioux City, IA) and water.[8] As the gel to water ratio increases it is expected that the material will become more solid and stiff. The gels were cast in rectangular 10cm^3^ molds and allowed to set at 4˚C for 12 hours. The mass of each gel was measured and the amount was adjusted (depending on the gel to water ratio) to ensure that the mass of each gel was 10g. Each gel was molded around a small aluminum jig, sized 5 cm x 5 cm x 2.5 cm, on which a piezoelectric accelerometer (model 352C33, PCB electronics, Depew, NY—0.5 Hz to 10 kHz frequency range) was mounted. The mold was removed, ensuring that the gel was the only mass above the accelerometer. To characterize the biomechanical parameters of the gels using the piezoelectric accelerometer, the gel was raised 5 cm off a rigid table and released; on landing, oscillations along the vertical axis were measured by the accelerometer. Each test consisted of five trials. The reproducibility of this manner of testing was assured by examining the variability of measured responses. Biomechanical values obtained by the accelerometer were compared to the MyotonPRO by leaving the mounting jig in place during subsequent tests and leaving the gels in the moulds, so as to measure the oscillation values of the same collective system in both tests. The MyotonPRO was programmed to automatically take 5 measurements, each separated by 2 seconds to allow for the oscillations in the gel to dissipate before the next test began, with a tap time of 15ms.

Based on the accelerometer output, the same mechanical and viscoelastic properties displayed by the MyotonPRO were derived [4] as follows: The period T, was defined as the time elapsed between the first two adjacent acceleration peaks following the mechanical impulse. This differs slightly from Schneider et al,[4] where T was defined as time elapsed between two adjacent zeroes. The oscillation frequency *f* was calculated from its definition:

f=1/T (1)

We assume an initial undamped vibration, in which case the angular frequency ω is related to stiffness K and mass m:

$$\omega =\sqrt{K/m}$$ (2)

Rearranging (2):

$$K=\omega^{2}m$$ (3)

The definition of the angular frequency is:

ω=2πf (4)

Substituting (4) into (3):

$$K=4\pi^{2}f^{2}m$$ (5)

where *f* is found from the measured period T using (1) and m is the mass of the gel above the accelerometer in kilograms. The latter equation was used, rather than K = am/d as in Schneider et al,[4] (where a is acceleration) to avoid the requirement of calibrating any zero offset for an accurate absolute value of peak acceleration. Finally, the decrement was defined as the natural log of the ratio between two adjacent acceleration peaks.

$$D=ln(a_{1}/a_{2})$$

where a_2_ is the peak successive to a_1,_ as determined from the voltage peaks of the accelerometer.

### Effect of overlying skin and subcutaneous tissue

Following this initial study, we examined the potential effect of the layer of skin and subcutaneous tissue which lie directly over the tissues whose properties are to be measured by the MyotonPRO. Two fresh (unfrozen) whole chickens were commercially obtained, and the bilateral Achilles tendons were used (n = 4 tendons in total). Before testing, the samples were marked on the skin using a felt-tipped pen in one centimeter increments, starting from the calcaneal bone and moving proximally to define a set of consistent probing sights. These sites coincided with measuring directly over the Achilles tendon (site 1), where the tendon begins branching towards the medial and lateral gastrocnemius muscles (site 2), where the tendon begins inserting into the muscles (site 3), continues inserting (site 4), finishes inserting (site 5), and finally the last two points were over the gastrocnemius muscles themselves (sites 6 and 7). At each site, 5 measurements were taken before moving to the next. The default testing pattern settings of the device were used with a 0.8 second interval between taps and a tap time of 15 ms. The layers of skin and subcutaneous tissue were removed afterwards in a single layer using a surgical scalpel, and the thickness of this skin and associated tissue was measured with digital calipers (Mastercraft #58-6800-4, Canadian Tire, Toronto, Canada). The same testing sites were then re-measured directly on the Achilles tendon or its associated muscle. The anteroposterior thickness of the Achilles tendon was also measured 1 and 2 cm proximal to its insertion with digital calipers.

### Reliability in human subjects

10 healthy adults (5 men and 5 women, aged 20–28) were recruited to examine the same-day test-retest reliability of the MyotonPRO with reference to the Achilles and patellar tendons. To be included, subjects had to be 19 to 50 years of age, fluent in English, and recreationally active. Subjects were excluded if they had any health or medical conditions which could affect the patellar tendon. All subjects provided written, informed consent. The height and weight of subjects were measured, and they were asked how many days / week of moderate to vigourous activity they engage in. To measure the stiffness of the patellar tendon, subjects were seated in long sitting (legs outstretched and relaxed), and the device was positioned perpendicular to the skin in the middle of the patellar tendon by manually palpating the insertions and lateral borders of the tendons. To measure the stiffness of the Achilles tendon, subjects were seated on a raised stool with the leg and foot hanging freely (hip and knee at approximately 90° of flexion). The testing protocol (number and spacing of taps) was identical to that used for the in vitro study. For each tendon, two measurement sessions were held on the same day with the same tester, separated by a minimum of 3 hours.

### Field study

Sixy-six recreational runners (40 men and 26 women, aged 20–75) were recruited at community-organized races in the Vancouver area during the summer of 2016. A booth with information about the study was placed near the starting line, and investigators recruited participants who approached the booth. All participants provided written, informed consent for measurement before and after the start of the race. A single investigator took bilateral measures of tendon stiffness before and after the race, using the same device, and using the protocol described in the reliability study described above. Participants provided the following information; height and weight (self-reported), average number of running days per week, average weekly miles run, and current medications (over last 48 hrs). To be included, participants had to be 18 years of age or older and fluent in English, participate in moderate or vigorous physical activity for a minimum of 30 minutes at least three times per week, and already be signed up to participate in the race. Exclusion criteria were: genetic disorders with known or possible influence on musculoskeletal properties; recently (within 12 months) experienced or currently have acute or chronic musculoskeletal disorders; recently (within 12 months) experienced a partial or full tear of the Achilles or patellar tendons.

### Statistical analysis

Descriptive statistics (mean, SD) were generated to summarize the measurements of ballistics gel obtained with the MyotonPRO and the external reference device. To determine if there was a statistically significant difference in the measured stiffness of avian tendons with and without the presence of skin, a three-way within subject ANOVA test was conducted with the following effects: (1) skin/no skin, (2) testing site, and (3) left/right leg. To examine the reliability of using the MyotonPRO for assessing the biomechanical properties of the human Achilles and patellar tendons, intraclass correlation coefficients were calculated for each dependent variable. To see if baseline Achilles tendon stiffness correlated with the type of race being run (as an indirect indicator of training status), a Spearman’s correlation coefficient was calculated. To examine for potential changes in Achilles tendon stiffness following running, a repeat measures ANOVA was run. Finally, to determine whether tendon stiffness differed between men and women at baseline, an independent t-test was performed (including both patellar and Achilles tendons).

## Results

### Examination of biomechanical parameters

The output of the MyotonPRO was compared to the values obtained through external measurement with a piezoelectric accelerometer, with regard to two biomechanical parameters of interest (K and D). The results from both testing methods for gels of varying consistencies are shown in Table 1. As expected, gels with increasing concentration of gelatin exhibited increasing stiffness and frequency. Despite the fact that our experimental set-up necessitated a slightly different method of calculation than that used by the MyotonPRO,[4] nevertheless the results obtained with the external measurement were remarkably consistent with the values obtained by the MyotonPRO for all three variables, with the mean values from the MyotonPRO falling within the standard deviation. The standard deviation of the MyotonPRO was smaller than that of the external reference.

**Table 1:** Mechanical properties measured by the MyotonPRO device compared to an external reference accelerometer.

	REFERENCE		MYOTONPRO	
GEL:WATER MIXTURE RATIOS	K (N/m)	D	K (N/m)	D
**1:30**	121 ±15	0.85 ±0.06	123 ±1	0.89 ±0.00
**1:20**	250 ±11	0.83 ±0.04	244 ±4	0.81 ±0.02
**1:10**	385 ±17	0.85 ±0.03	388 ±6	0.88 ±0.01

Data are shown as mean +/- SD. K = stiffness, D = decrement.

### Effect of overlying skin and subcutaneous tissue

The skin thickness over the avian Achilles tendon was consistent, measuring 1.2 +/- 0.2 mm without significant variation across all 7 test sites. Achilles tendon thickness measured 5.1 +/- 0.2 mm, which is identical to the thickness of the asymptomatic, adult human Achilles tendon. [9] Whilst the MyotonPRO showed comparable measurements between tests with and without skin (Table 2), statistically significant differences were found for stiffness (p<0.001), which was on average 2.5% less stiff with the skin removed, and for decrement (p<0.001). It was also noted that the standard deviations in the experiments on avian tendons were larger than the MyotonPRO achieved on ballistics gel, but comparable with or without skin. There was a consistent trend in the measurements indicating the muscle-tendon complex became progressively stiffer from proximal to distal (S1 Table, S1 Fig).

**Table 2:** MyotonPRO output averaged over all testing sites.

	STIFFNESS (N/m)		DECREMENT	
**LEG**	Left	Right	Left	Right
**SKIN**	652 ±82	670 ±45	2.31 ±0.31	2.10 ±0.32
**NO SKIN**	632 ±74	656 ±42	2.45 ±0.46	2.30 ±0.38

Data are shown as mean +/- SD.

### Reliability in healthy adults

The average age of participants was 22.2+/-2.7 years. BMI was 22.5+/-2.9 kg.m^2^, and average number of days engaged in exercise per week was 2.0 +/-2.1. For the Achilles tendon, the reliability (intra class correlation coefficient) of the measured parameters were: frequency (0.96), decrement (0.94), stiffness (0.96). The intraclass correlation coefficients for the patellar tendon were: frequency (0.96), decrement (0.86), stiffness (0.96).

### Examination of trends in healthy runners

The average age of participants was 42 years ± 14, BMI 23 ± 2.3. From the demographic information presented by race-type (S2 Table), it can be seen that marathon runners trained more intensively and were slightly older than the half-marathon or 10 km runners, but were equivalent in BMI and male/female ratio. The average baseline stiffness of the Achilles tendon was weakly correlated with the type of race (r = 0.184, p = 0.035), with a progressive increase in stiffness as race distance increased. The stiffness of the Achilles after running was noted to experience on average a small increase compared to before running across the entire group (p = 0.052). Men’s tendons tested as significantly stiffer than women’s (p = 0.012)

## Discussion

The MyotonPRO, when tested on soft-tissue phantom materials with known properties, was capable of quantifying dynamic stiffness (K), and logarithmic decrement (D). The device was capable of acquiring values across the range gels tested, as can be seen in Table 2. The subjects in the study by Schneider et al.[4] were slightly older, and the Achilles tendon stiffness was slightly higher than that measured in the current study, which is in keeping with the known age-related increase in tendon stiffness among healthy adults.[10] The reliability of most of the biomechanical measures in the human tendons tested (Achilles and patellar) was adequately high (ICC>0.90) such that these devices could potentially be useful in monitoring individual changes in tendon biomechanical properties, e.g. in clients undergoing rehabilitation for tendon problems or engaging in preventative exercise aimed at strengthening tendons.

While there was a statistically significant difference in stiffness between measurements made with or without the skin overlying the tendon and/or muscle, the difference was small and consistent across the measured sites (approximately 2.5%), such that the device was still able to detect changes in stiffness along the length of the muscle-tendon unit even in the presence of skin. We do not know why the inclusion of skin resulted in a slight increase in stiffness values. Nevertheless, the influence of skin did not have a meaningful impact on the measured value, at least in the in vitro study performed here. Changes in (tensile) stiffness of the Achilles tendon following acute resistance training programs are in the order of +30%, [11] and loss of stiffness in tendinopathic tendons is on average -20%.[12] Further testing could be done to determine if there is a cut-off value for the thickness of skin beyond which the measurement is no longer valid. In addition, the stiffness of skin may vary considerably from person to person and over different anatomical regions. One limitation of this aspect of the study is that it was conducted using an avian model. Although there are some superficial similarities of the avian tendon and skin to humans (e.g. similar skin and tendon thickness), there are differences as well. The human tendons were stiffer than the avian tendons, which is teleologically in keeping with the comparatively higher loads placed on human tendons. The commercially obtained animals used in this study were observed (qualitatively) to be very uniform in size and composition (average weight 1.7 kg, SD 0.07 kg), which is in stark contrast to humans whose size, BMI, and extent of subcutaneous fat vary substantially. Future studies, perhaps using calipers or greyscale ultrasound, could examine a wider range of subcutaneous tissue thickness.

The field confirmed a number of previously reported findings, e.g. stiffer tendons in men than women. There was a small increase in Achilles tendon stiffness after running of longer duration. The type of race (marathon, half-marathon or 10 km) was significantly (but weakly) correlated with Achilles tendon stiffness. This could indicate either an effect of training (marathon runners reported greater frequency and length of running), or of age (marathon runners were slightly older–please see S2 Table). A limitation of this study was the relatively small number of runners in each race category, which means that the possible training effect is obscured by other factors such as age. In addition, a laboratory-based study would have been able to better control the conditions of running, e.g. speed and incline. The majority of all three race types occurred on flat, pavemented road. Future laboratory studies could provide more insight into the time course and the influence of speed and incline on tendon stiffness, and the impact of tendon stiffness on running performance.

## Conclusion

The MyotonPRO provided results for all measurement parameters with a very small margin of error when tested against materials with known properties. The effect of skin on the device output was statistically significant, but it did not interfere with the trends observed in the mechanical properties along the tissue length, and did not interfere with the ability to detect physiologically expected variation in Achilles tendon properties in a group of healthy adult runners, including chronic influences such as the effect of sex/gender, training status, and acute influences (i.e. post-race changes in stiffness). The reliability of measurements from the patellar and Achilles tendons in a small group of healthy young adults was excellent. Further work is required to discover normal values in a larger population of healthy and pathological tendons, and with individuals of varying skin type and body composition.

## Practical implications

- Tendon mechanical properties can be quantified with the MyotonPRO device.
- In individuals with normal BMI, the trends in, and region-specific properties of, the Achilles and patellar tendons can be monitored for relative changes with the MyotonPRO device.
- Future studies are needed to determine the utility of the MyotonPRO in people with higher levels of subcutaneous adiposity.

## Supporting information

S1 Fig Stiffness response over testing sites (left leg). Error bars represent the standard deviation.
(DOCX)

S1 Table MyotonPRO output showing difference in tendon (site 1) and muscle (site 7) properties. Data are shown as mean +/- SD. Tendon and muscle were significantly different for all measured parameters.
(DOCX)

S2 Table Characteristics of recreational runners participating in field study. BMI: body mass index.
(DOCX)
