# Supplementary material for: Feasibility of using a hand-held device to characterize tendon tissue biomechanics
Source: PLoS One. 2017 Sep 6;12(9):e0184463. doi: 10.1371/journal.pone.0184463 (PMC5587276; doi:10.1371/journal.pone.0184463)
Supplement: S1 Fig — Error bars represent the standard deviation. (DOCX) [file pone.0184463.s001.docx]

**S1 Figure**
