# Supplementary material for: Feasibility of using a hand-held device to characterize tendon tissue biomechanics
Source: PLoS One. 2017 Sep 6;12(9):e0184463. doi: 10.1371/journal.pone.0184463 (PMC5587276; doi:10.1371/journal.pone.0184463)
Supplement: S1 Table — Data are shown as mean +/- SD. Tendon and muscle were significantly different for all measured parameters. (DOCX) [file pone.0184463.s002.docx]

**S1 Table. MyotonPRO output showing difference in tendon (site 1) and muscle (site 7) properties.**

|  | Frequency (Hz) | | Stiffness (N/m) | | Decrement | |  | |  | |
| --- | --- | --- | --- | --- | --- | --- | --- | --- | --- | --- |
|  | Skin | No skin | Skin | No skin | Skin | No skin |  |  |  |  |
| Tendon | 40.2 ±1.0 | 33.7±0.5 | 728 ±17 | 704±7 | 1.81 ±0.11 | 1.61 ±0.06 |  |  |  |  |
| muscle | 29.8±0.9 | 32.8 ±1.6 | 598±34 | 590 ±31 | 2.34 ±0.12 | 2.64 ±0.13 |  |  |  |  |

Data are shown as mean +/- SD. Tendon and muscle were significantly different for all measured parameters.
