# Supplementary material for: Feasibility of using a hand-held device to characterize tendon tissue biomechanics
Source: PLoS One. 2017 Sep 6;12(9):e0184463. doi: 10.1371/journal.pone.0184463 (PMC5587276; doi:10.1371/journal.pone.0184463)
Supplement: S2 Table — BMI: body mass index. (DOCX) [file pone.0184463.s003.docx]

**S2 Table. Characteristics of recreational runners participating in field study.**

| **Type of Race** | **Age (yr)** | **Sex (m/f)** | **BMI (kg/m2)** | **Days run / week** | **Km run/ week** |
| --- | --- | --- | --- | --- | --- |
| All | 42 ± 14 | 40 / 26 | 23 ± 2.3 | 3.5 ± 1.2 | 34 ± 23 |
| Marathon | 48 ± 13 | 12 / 7 | 23 ± 2.5 | 4.1 ± 1.1 | 53 ± 25 |
| Half-marathon | 38 ± 13 | 13 / 10 | 23 ± 2.0 | 3.0 ± 1.0 | 25 ±+ 13 |
| Ten km | 38 ± 15 | 15 / 9 | 23 ± 2.3 | 3.3 ± 1.0 | 25 ± 17 |

BMI: body mass index
